# MicroRNAs: New Insights into Chronic Childhood Diseases

**DOI:** 10.1155/2013/291826

**Published:** 2013-06-27

**Authors:** Ahmed Omran, Dalia Elimam, Fei Yin

**Affiliations:** ^1^Department of Pediatrics and Neonatology, Suez Canal University, Ismailia 41522, Egypt; ^2^Department of Pediatrics, Xiangya Hospital of Central South University, 87 Xiangya Road, Changsha, Hunan 410008, China

## Abstract

Chronic diseases are the major cause of morbidity and mortality worldwide and have shown increasing incidence rates among children in the last decades. Chronic illnesses in the pediatric population, even if well managed, affect social, psychological, and physical development and often limit education and active participation and increase the risk for health complications. The significant pediatric morbidity and mortality rates caused by chronic illnesses call for serious efforts toward better understanding of the pathogenesis of these disorders. Recent studies have shown the involvement of microRNAs (miRNAs) in various aspects of major pediatric chronic non-neoplastic diseases. This review focuses on the role of miRNAs in four major pediatric chronic diseases including bronchial asthma, diabetes mellitus, epilepsy and cystic fibrosis. We intend to emphasize the importance of miRNA-based research in combating these major disorders, as we believe this approach will result in novel therapies to aid securing normal development and to prevent disabilities in the pediatric population.

## 1. Introduction

The prevalence of children with chronic illnesses varies widely with an overall rate of 10% to 20% [1] and is expected to increase further. Childhood chronic illnesses represent a major challenge and burden for affected families and the health care system. There is evidence that chronically ill children and their families are at greater risk for developing psychological and emotional difficulties than healthy children and their families. Many chronically ill children grow up in hospitals and live a life far from normal due to recurrent hospitalizations. They often show growth retardation as a result of the illness itself or its pharmacological treatment options. The long-term requirement for medical and social care of these children can be extremely complex and expensive.

The mandate for the child to adopt many self-care skills for monitoring and safety represents a major part of the challenge during the disease course.

The main goal for pediatricians is to maximize the children's functional abilities and sense of well-being, their health-related quality of life, and their development into healthy and productive adults. Chronic diseases in children and adolescents are far from rare and are today more likely described as an epidemic, which calls for major efforts to understand causation and improve prevention and treatment protocols.

There is a wealth of evidence on the diverse role of miRNAs in many biological processes, including proliferation, differentiation, apoptosis, and development. The list of diseases in which dysregulation of miRNAs has been implicated is constantly growing and includes major pediatric chronic non-neoplastic diseases. We recently reviewed the role of miRNAs in pediatric central nervous system and cardiovascular diseases including congenital heart diseases [2, 3]. This review summarizes recent progress in edge-cutting research about the involvement of miRNAs in bronchial asthma, diabetes mellitus, epilepsy, and cystic fibrosis (Table 1).

## 2. miRNAs and Bronchial Asthma

Bronchial asthma is a chronic disorder of the airways that is characterized by variable and recurring airflow obstruction, chronic airway inflammation, bronchial hyperresponsiveness, and tissue remodeling [4, 5]. Three hundred million people are suffering from asthma worldwide, over 22 million people in the United States alone, of which over 6 million are children. The illness related cost is 6.2 billion US Dollars each year.

In the pediatric population, bronchial asthma is one of the most common chronic lung diseases affecting around 5%–10% of school-age children [6]; it is associated with reduced quality of life and exercise intolerance, accounts for a loss of 10 million school days [7], and is a leading cause of hospitalizations in children [8]. Current available asthma treatment is not effective in preventing airway remodeling processes and fails to prevent asthma exacerbations and hospitalizations even in children on appropriate controller medications.

An improved understanding of the molecular mechanisms in asthma through exploring the role of miRNAs is expected to create promising potentials to reveal new approaches for primary prevention and identification of new therapeutic targets in childhood asthma.

miRNAs appear to play an important role in asthma development and pathogenesis. Susceptibility to asthma has been linked to the variation in specific miRNA genes and/or their specific miRNAs. The 3′UTR of HLA-G, a gene, which has been identified as an asthma-susceptibility gene [9], was found to be targeted by miR-148a, miR-148b, and miR-152. The possibility that miRNA variation is a key factor in the risk of developing asthma has been further supported. Significant differences in the genotype and allelic distribution of the pre-miRNAs SNPrs2910164G/C and rs2292832C/T among asthmatics and their controls indicated that this SNP may play a role in asthma development [10]. Another study suggested that decreased expression level of miR-155 plays an important role in the development of asthma and is correlated to asthma disease severity as well [11].

Recent reviews show the involvement of miRNAs in both the immunological and inflammatory components of asthma pathogenesis as well as in the neuronal control of airway smooth muscles. The role of miRNAs in the regulation of immunological pathways in asthma pathogenesis is rather central. The first evidence was obtained through detecting abnormal expression levels of miRNAs in asthma, including miR-146b, miR-223, miR-29b, miR-29c, miR-483, miR-574-5p, miR-672, and miR-690 [12–19].

Extrinsic asthma is an IgE mediated hypersensitivity reaction where the bridging of IgE triggers the release of mast cell mediators. MiR-221 is a likely regulator of mast cell activation [20] and proliferation including mast cells differentiation, migration, adhesion, cytokine production, and survival upon withdrawal of essential cytokines [21].

Asthma is described as Th2 mediated inflammation of the airway [22, 23]. Th2 cells, which play a fundamental role in allergic asthma pathogenesis [12], are polarized by cytokine IL-12p35, the molecular target of miR-21.

Upregulation of miR-21 in the allergic airway indicates its involvement in inflammation. Of similar importance to the pathogenesis of allergic airways disease is miR-126 [13]. The blockade of miR-126 suppressed the asthmatic phenotype in the form of diminished Th2 responses, suppressed inflammation, reduced airway hyperresponsiveness (AHR), inhibited eosinophil recruitment, and lowered mucus secretion [13].

IL-13 induces asthma features, such as epithelial cell hyperplasia, goblet cell metaplasia, deposition of various extracellular matrix proteins in subepithelial regions, and increased airway smooth muscle cell contractility, and seems to be under miRNA control [24]. 

miR-146a mimics modulate human bronchial epithelial cells (HBEC) survival by upregulating Bcl-XL and STAT3 phosphorylation and appear thereby to contribute to processes of tissue repair and remodeling which are hallmarks in asthma pathogenesis [25].

Intranasal administration of let-7 mimic reduces IL-13 levels in allergic lungs and alleviates these features [26], indicating that let-7 has anti-inflammatory effect through reduction of IL-13. 

MiR-145 demonstrated to play an additional central proinflammatory role in the development allergic airways inflammation to house dust mites [27]. 

In addition to inflammation, dysfunctional neural control of airway smooth muscles (ASMs) is a major component of asthma pathogenesis. A functional cascade that involves Sonic hedgehog (Shh), miR-206 and brain derived neurotrophic factor (BDNF) has been recently uncovered and found to coordinate ASM formation and innervations [28]. Sonic hedgehog signaling blocks miR-206 expression, which results in increased BDNF protein expression. 

Bronchial epithelium is a major source of many key inflammatory and remodeling molecules [29–32]. These stimulated bronchial epithelial cells with TNF-*α* and IL-4 revealed that let-7, miR-29a, and miR-155 have been involved in the regulation of allergic inflammation [33].

MiR-133a negatively regulates RhoA in bronchial smooth muscle cells (BSMCs), a new target for asthma therapy [17]. Furthermore, downregulated miR-133a by IL-13 in the BSMCs causes an upregulation of RhoA, presumably resulting in an augmentation of bronchial smooth muscle contraction [34].

miRNAs appear to be attractive new drug targets. Th2-driven airway inflammation, mucus hypersecretion, and AHR were shown effectively suppressed by delivery of an antagomir that inhibits miR-126 [13]. Recently, miR-106a was demonstrated to inhibit IL-10 in the posttranscriptional phase, which significantly alleviated most of the features of asthma. This represents the first in vivo proof of a miRNA- mediated regulation of IL-10 with a potential to reverse an established asthmatic condition [35].

Glucocorticoids are used as mainstay therapy for asthma. In a murine asthma model, reported downregulation of miR-146a as an effect of dexamethasone, might partially explain its anti-inflammatory mechanism [36]. Antagonizing the function of miR-145 was as effective as glucocorticoid therapy in a trial treating mice treated with anti-miR-145 or dexamethasone and displayed significant reduction in the severity of the inflammatory lesions induced by HDM challenge [27]. The RhoA/Rhokinase pathway has now been proposed as a new target for the treatment of AHR in asthma [37, 38] and modulation of this pathway by miR-133a might provide a new insight into the treatment of AHR [17].

## 3. miRNAs and Diabetes Mellitus (DM)

Diabetes is one of the most common chronic diseases in the world and is recognized as one of the most important health threats of our time. DM is associated with serious morbidity and chronic disabling complications attributing to its high rate of mortality. Both type 1 (T1D) and type 2 diabetes mellitus (T2D) occur in children. T1D is a chronic autoimmune disease with an increasing incidence in the European pediatric population [39]. T2D, previously considered an adulthood disease, has now an increasing prevalence of early onset T2D secondary to the childhood obesity pandemic [40].

New approaches in investigating diabetes are essential for a deeper understanding of its pathogenesis and for developing novel therapeutic strategies. In recent years, miRNAs have become one of the most encouraging and fruitful fields in biological research and have been implicated as new players in the pathogenesis of diabetes and diabetes-associated complications. 

The role of miRNAs in DM starts as early as the development of pancreatic islets. MiR-124a2 and miR-375 are involved in pancreatic beta-cell development [41, 42] and are necessary for proper formation of pancreatic islets in vertebrates. MiR-375 is necessary for the development of *β*-cells in mice [42], establishment of normal pancreatic endocrine cell mass in the postnatal period, and maintenance of its viability [43]. Loss of miR-375 results in pancreatic cell defect and chronic hyperglycemia.

miRNAs have been further shown to regulate various physiological events relevant to DM pathophysiology, such as insulin biosynthesis, insulin secretion, insulin action, insulin responsiveness, and energy homeostasis.

miRNAs regulating insulin biosynthesis include miR-15a [44], miR-30d [45], miR-375, miR-122, miR-127-3p, and miR-184 [46]. MiR-15a increases insulin biosynthesis by targeting UCP-2 [44]. MiR-30d increases MafA expression, which promotes the transcription of the insulin gene in pancreatic *β*-cells [45]. MiR-375, miR-122, miR-127-3p, and miR-184 are suggested to play an important role in *β*-cell function insulin biosynthesis [46]. Suppression of human islet insulin biosynthesis by high glucose has been demonstrated to be induced by miR-133a decreasing polypyrimidine tract binding protein expression [47].

MiR-9 was found to play a critical role in the control of the secretory function of insulin-producing cells [48, 49]. 

MiR-375 is the highest expressed miRNA in pancreatic islets of humans and mice and regulates insulin secretion in isolated pancreatic cells [50]. Overexpression of miR-375 reduces insulin secretion through inhibition of exocytosis of insulin granules via translational repression of the cytoplasmic protein myotrophin [50]. Mice lacking miR-375 (375KO) are hyperglycemic and pancreatic *β*-cell mass is decreased due to impaired proliferation [43]. Li et al. (2010) showed also that miR-375 enhanced palmitate-induced lipo-apoptosis in insulin-secreting NIT-1 cells by repressing myotrophin (V1) protein expression [51]. Optimal insulin secretion in *β*-cells requires additional appropriate levels of miR-124a, miR-29 [41, 52], and miR-33a. MiR-33a was just recently shown to affect insulin secretion and acts through regulating its expression to correlate inversely with the expression of ABCA1 in pancreatic islets [53]. MiR-21, miR-34a, and miR-146 were shown to function as negative regulators of insulin signaling via inhibition of insulin secretion [54].

Recently, studies have shown the role of miRNAs in insulin sensitivity with emphasis on the importance of miR-103/107 [55]. The Lin28/let-7 pathway is a central regulator of mammalian glucose metabolism through interactions with the insulin-PI3 K-mTOR pathway and T2D-associated genes [56].

T1D, insulin dependent diabetes mellitus (IDDM), is a chronic autoimmune disorder caused by the interaction of environmental factors with an inherited predisposition. Twenty-seven miRNAs were mapped and located in 9 T1D susceptibility regions, rendering these miRNAs candidates for T1D susceptibility genes [57]. 

Regulatory T cells (Tregs) are known critical regulators of autoimmune diseases, including T1D. miRNA expression profiles in Tregs of T1D patients revealed a significant higher expression of miR-146a and lower expression miR-20b, miR-31, miR-99a, miR-100, miR-125b, miR-151, miR-335, and miR-365 [58]. These results support the hypothesis that changing expression in specific miRNAs can influence the function of Tregs and therefore the pathogenesis of T1D.

During the initial phases of T1D, immune cells invade pancreatic islets, exposing *β*-cells to pro-inflammatory cytokines. Cytokine-mediated *β*-cell dysfunction is suggested to be modulated by miR-29, which appeared to be dysregulated in this phase [59]. MiR-326 is expressed at higher levels in T1D subjects with ongoing islet autoimmunity [60]. miRNA array profiling in a T1D model identified eight miRNAs (miR-124, miR-128, miR-192, miR-194, miR-204, miR-375, miR-672, and miR-708) with differential expression that are likely involved in *β*-cell regulatory networks [61].

Dicer studies provide clear evidences for its role in the T1D pathogenesis. *β*-cells specific Dicer1 deletion results in aberrant pancreas development and neonatal death [62] and its inactivation leads to development of diabetes due to reduced insulin expression [63]. Targeted disruption of the Dicer1 gene specifically in *β*-cells leads to progressive reduction in insulin secretion and glucose tolerance and development of diabetes [64].

MiRNAs are also emerging as highly tissue and/or cell-specific biomarkers of autoimmunity in T1D. The possibility of measuring miRNA in body fluids such as serum would help to easily recognize these particular markers [65].

T2D is a major health issue that has reached an epidemic status worldwide and is tightly linked to obesity. Obesity is characterized by intracellular accumulation of lipid in the pancreatic islets leading to *β*-cellular dysfunction and ultimately contributes to the pathogenesis of T2D [66, 67]. T2D is a progressive metabolic disorder characterized by reduced insulin sensitivity, insulin resistance and pancreatic *β*-cell dysfunction.

A growing body of direct evidence implicates the role miRNAs in T2D and most of its pathophysiological aspects. Recent experiments provide direct evidence that obesity induces overexpression of miR-143, which acts to inhibit insulin-stimulated AKT activation leading to impairment of glucose metabolism [68].

Subclinical inflammation and insulin resistance implicated in T2D patients are a result of impaired function of miR-146a and its downstream signals [69].

MiR-125a was found to be over-expressed in insulin target tissues in a spontaneous rat model of T2D [70]. MiR-125a is suggested to contribute to insulin resistance and play a critical role in insulin signaling [71] through affecting genes involved in the MAPK signaling pathway implicated in T2D [72].

Seven diabetes-related serum miRNAs: miR-9, miR-29a, miR-30d, miR-34a, miR-124a, miR-146a, and miR-375 [73], had been reported previously as key gene regulators involved in the regulation of insulin gene expression, insulin secretion [41, 43, 48], insulin signaling in target tissues [74], and free fatty acid (FFA) mediated *β*-cell dysfunction [75], all of which are closely related to the pathogenesis of T2D.

Deregulated miRNAs associated with T2D were identified as useful distinguishing serum biomarkers for different stages of diabetes progression and include miR-144, miR-146a, miR-150, miR-182, miR-192, miR-30d and miR-320. The expression profiles of these miRNAs can differentiate between impaired fasting glucose state (IFG) and well-developed T2D [76]. The first evidence that plasma miRNAs are deregulated in patients with DM was obtained from the observation that endothelial miR-126 was lost in type 2 diabetic patients [77].

Both T1D and T2D can lead to debilitating microvascular complications such as retinopathy, nephropathy, and neuropathy, as well as macrovascular complications. 

A significant association between altered miRNA expression and the development and progression of the various diabetes complications has been recently reported. Several studies have demonstrated a role for miRNAs in diabetic nephropathy (DN) and was first demonstrated by Kato et al. in 2007. The authors found increased expression of miR-192 in glomeruli from mice with both type 1 and type 2 diabetes as well as in TGF-*β* treated cultured mesangial cells (MCs) [78]. TGF-*β* signaling events are crucial in regulating fibrotic effects in MCs and other renal cells through subtle molecular mechanisms that are yet not fully clear.

Of particular interest is a group of miRNAs including miR-200b/c, miR-216a, and miR-217, which were found to be upregulated in mouse renal mesangial cells (MMC) treated with TGF-*β* and in glomeruli of mouse models for diabetes [79–81]. These key miRNAs are highly expressed in the kidney and can act as effectors of TGF-*β* actions and high glucose in diabetic kidney disease.

Renal fibrosis is a component of DN and it was found that miR-377 induces fibronectin (ECM protein) expression in MCs via downregulation of manganese superoxide dismutase and p21 activated kinase indicating its role in pathogenesis of microvascular complications [82]. Specific reduction of renal miR-192 on the other hand decreases renal fibrosis and improves proteinuria, lending support for the possibility of an anti-miRNA-based translational approach to the treatment of DN [83]. 

Diabetic retinopathy (DR) is one of the leading causes of blindness. miRNAs are involved in the pathogenesis of DR through the modulation of multiple pathogenetic pathways and may be novel therapeutic targets for the treatment of DR [84–86]. 

Diabetic individuals are two to four times more likely to have vascular and heart disease compared to the normal population, and 75% of diabetes related deaths are due to heart diseases. Cardiac involvement in diabetes includes coronary atherosclerosis, diabetic cardiomyopathy, and autonomic neuropathy.

Accumulating evidence suggests that miRNAs are involved in the process of angiogenesis by modulating new vessel formation through their upregulation or downregulation [87, 88]. Among downregulated miRNAs in DM patients, miR-126, miR-27b, and miR-130a have been identified as proangiogenic miRNAs [89].

Tribble 2 (TRB2) plays important roles in the pathogenesis of T2D large artery complications at early stage and seems to be modulated by miR-98. Thus, targeting TRB2 and miR-98 could be considered as novel therapeutic strategies for T2D early large artery complication [90].

Caporali et al. have augmented our understanding of miRNA biology in vascular pathophysiology in diabetic patients through detecting the causal role of miR-503 in diabetes-induced impairment of endothelial function and reparative angiogenesis [91]. MiR-126 downregulation in endothelial progenitor cells (EPC) from diabetes patients leads to impairment in their functions via targeting gene Spred-1 [92].

Many miRNAs are promising to have a future role in the development of treatments of DM. Human embryonic stem (hES) cells have proven to possess unlimited self-renewal and pluripotency and thus have the potential to provide an unlimited supply of different cell types for tissue replacement. Hence, hES cells are considered in the effort to find replacement for damaged islet *β*-cells especially T3 cells (T3pi). 

Pancreatic islet-like cell clusters derived from T3 cells showed very high expression of miRNAs including miR-186, miR-199a, and miR-339, which downregulate the expression of LIN28, PRDM1, CALB1, GCNT2, RBM47, PLEKHH1, RBPMS2 and PAK6. Therefore, manipulation of these miRNAs may be useful to increase the proportion of beta cells and insulin synthesis in the differentiated T3pi for cell therapy of TID [93].

A unique regulatory pathway of *β*-cell death involves miR-21. MiR-21 targets the tumor suppressor gene PDCD4 and its upstream transcriptional activator nuclear factor-*κ*B (NF-*κ*B); thus, targeting the miR-21−PDCD4 pathway may represent a unique strategy for treating autoimmune T1D [94].

As reported previously, miR-375 negatively regulates insulin secretion, and attenuation of miR-375 through the cAMP-PKA pathway may facilitate the insulin response in pancreatic *β*-cells [53].

Sirtuin-1 (SIRT1) is a potential therapeutic target to combat insulin resistance and T2D [95]. SIRT1 is regulated by miR-181a and improves hepatic insulin sensitivity. Inhibiting miR-181a might be a potential new strategy for treating insulin resistance and T2D [96].

Islet transplantation represents a potentially interesting strategy for T1D therapy. However, allogeneic islet grafts require immunosuppressive therapy to avoid rejection. Therefore, immune system modulation is necessary for functional stabilization of the transplantation. Adequate knowledge of the role of miRNAs in the regulation of immune function could result also in the possibility to design a novel immunosuppressive therapy for pancreatic islet transplantation.

## 4. miRNAs and Epilepsy

Epileptic disorders are serious chronic brain disorders among the most frequent neurologic problems that occur in childhood. Approximately 2% of the population is affected by epilepsy (lifetime prevalence), and in the majority (three-fourths), the onset of epilepsy occurs in the pediatric age group. At least 12% of patients with childhood-onset epilepsy will have a period of intractability during long-term followup [97], for which epilepsy surgery has become an increasing treatment option [98]. Children with seizures are at increased risk for mental health impairments, developmental and physical comorbidities, increasing needs for care coordination, and specialized services [99].

Attention has been recently drawn to the role of miRNAs in pediatric CNS diseases [2], including epilepsy, shedding new light on the molecular mechanism promising novel therapeutic targets and effective antiepileptogenic medications.

Loss of Dicer in neurons or astrocytes results in miRNA downregulation, neuronal dysfunction, apoptosis, seizures, and cognitive deficits [100]. This observation was confirmed by a study showing reduced mature miRNAs levels in the human temporal lobe epilepsy (TLE) as a result of Dicer loss [101]. These findings suggest that loss of Dicer and failure of mature miRNA expression may be a feature of the pathophysiology of hippocampal sclerosis (HS) in patients with TLE and future efforts might be directed to determining whether restitution of Dicer to such tissue restores mature miRNA production and influences the epileptic phenotype.

Status epilepticus (SE) induces a cascade of molecular changes that contribute to the development of epilepsy. In the acute stage of mesial temporal lobe epilepsy (MTLE) development in rats, 19 miRNAs were up-regulated, amongst which miR-213, miR-132, miR-30c, miR-26a, and miR-375 were the most prominent upregulated miRNAs. Seven miRNAs were downregulated including miR-29a and miR-181c [102]. Neurotrophin-3 (NT-3) is a neurotrophic factor that has been implicated in the development of epilepsy in several rodent models. MiR-21 was identified as a candidate for regulating neurotrophin-3 signaling in the hippocampus following SE suggesting that miR-21 downregulates NT-3 which is responsible for increased neuronal cell loss following SE [103]. MiR-21 is also upregulated in children with MTLE [104].

Deregulated miRNAs may be involved directly or indirectly in the pathogenesis in both the acute and chronic stages of MTLE. One hundred and twenty-five miRNAs have been identified in the hippocampus of lithium-pilocarpine-induced TLE and normal rats, including 23 miRNAs that were expressed differentially in the chronic stage of MTLE development. Five miRNAs were found downregulated and include miR-let-7e. Eighteen miRNAs were found upregulated and include miR-23 a/b [105].

The role of neuroinflammation is emerging as a key element in the pathogenesis of MTLE, the most common form of partial-onset epilepsies that usually begins in childhood. Aronica et al. were the first to report an altered expression pattern of miR-146a associated with inflammation in epileptic rats and TLE patients, adding a new insight to molecular mechanisms in proepileptogenic inflammatory signaling processes [106]. MiR-146a and interleukin-1*β* (IL-1*β*) are differently expressed in the various stages of MTLE development in an immature rat model and in children. The different expression pattern of both IL-1*β* and miR-146a at various stages suggests an interactive relationship. Consequently, modulation of the IL-1*β*-miR-146a axis may be a new target for antiepileptic therapy [107]. Furthermore, we just very recently found that miR-155 and tumor necrosis factor alpha (TNF-*α*) showed the same pattern of expressions in the three stages of MTLE development in immature rat model and are upregulated in children with MTLE. We found also a direct relationship between them on the astrocyte level [108]. 

A genome-wide miRNA profiling study revealed segregated miRNA signatures and deregulation of 165 miRNAs in MTLE patients. The immune response was most prominently targeted by the deregulated miR-221 and miR-222. These miRNAs regulate endogenous ICAM1 expression and were selectively coexpressed with ICAM1 in astrocytes in MTLE patients, which suggest that miRNA changes in MTLE patients affect the expression of immunomodulatory proteins facilitating the immune response [109].

Increasing evidences highlight the role of synaptic plasticity in the development of MTLE [110, 111]. Recently miRNAs have been proposed to target neuronal mRNAs localized near the synapse, exerting a pivotal role in modulating local protein synthesis, and presumably affecting adaptive mechanisms such as synaptic plasticity [112, 113]. Using an in vivo model for increasing excitatory activity in the cortex and the hippocampus indicates that the distribution of some miRNAs can be modulated by enhanced neuronal (epileptogenic) activity. 

The dynamic modulation in the local distribution of miRNAs seems to play key roles in controlling localized protein synthesis at the synapse [114]. Pilocarpine-induced seizures led to a robust, rapid, and transient increase in the primary transcript of miR-132 (pri-miR-132) followed by a subsequent rise in mature miR-132 indicating that miR-132 is an activity-dependent in vivo, and may contribute to the long-lasting proteomic changes required for neuronal plasticity [115]. 

Taking a step in using miRNAa as blood biomarkers for epilepsy, Liu et al. described a unique expression of blood miRNAs 24 hours after induction of kainate seizures [116]. Also Hu et al. demonstrated a possible correlation between hippocampal and peripheral blood miRNAs in post-SE rats, through detecting similar expression patterns in miR-34a, miR-22, and miR-125a (upregulated) while miR-21 had decreased [102]. 

Very recently, in vivo microinjection of locked nucleic acid-modified oligonucleotides depleted hippocampal miR-132 levels and reduced seizure-induced neuronal death, thus strongly suggesting that miRNAs are important regulators of seizure-induced neuronal death [117]. We found in our study that brain-specific miR-124 and miR-134 were upregulated in the seizure related stages of MTLE in immature rat model and children with MTLE, suggesting that downregulation of these miRNAs may have anti-convulsive effects [104]. It was demonstrated additionally that silencing miR-134 exerts prolonged seizure-suppressant and neuroprotective actions giving promising hope for miRNAs to be useful as potential therapeutic target for epilepsy treatment [118]. Whether anti-miRNAs could function as anticonvulsants or would be true antiepileptogenic requires more experimental work.

## 5. miRNAs and Cystic Fibrosis

Cystic fibrosis (CF) is the most common lethal genetic disease in the Caucasian populations and occurs in approximately 1 in 2500 births [119]. It is caused by mutations in cystic fibrosis transmembrane conductance regulator (CFTR) gene. The most frequent mutation is deletion of a phenylalanine residue at position 508 (ΔF508).

The life expectancy of patients with CF has dramatically increased over the past decades [120], and the median survival of patients born in 2000 is expected to be above 50 years [121]. Despite significant advances in treatment regimes, CF remains a condition for which no effective cure exists and still has a mortality rate of >90% as a result of respiratory failure [122].

Investigating the expression and function of miRNAs in CF will shed light on previously unidentified regulatory mechanisms and further direct the development of future therapeutic strategies.

Emerging evidence suggests that changes in miRNAs expression are associated with CF [123–126]. It is hypothesized that unique miRNA expression profiles exist in CF versus non-CF bronchial epithelial cells and that these differential molecular miRNA signatures can regulate pro-inflammatory gene expression [124].

To date, several groups have examined the potential role of miRNAs in molecular pathways involved in the pathogenesis of CF, especially lung inflammation [127, 128]. MiR-155 is suggested playing an important role in the activation of IL-8-dependent inflammation in CF [126].

Several studies demonstrate that miRNAs regulate expression of the CFTR gene post transcriptionally. MiR-138 was discovered to regulate CFTR expression through its interaction with the transcriptional regulatory protein SIN3A. Treating airway epithelia with an miR-138 mimic indeed increased CFTR mRNA and enhanced CFTR abundance and transepithelial Cl (−) permeability independent of elevated mRNA levels. Anti-miR-138 had the opposite effects [129]. 

A role of miRNAs in targeting CFTR has been supported. hsa-miR-384, hsa-miR-494 and hsa-miR-1246 are involved in the post-transcriptional regulation of the CFTR channel synthesis. In individuals carrying the DF508 CFTR mutation, increased expression of miR-145, miR-223, and miR-494 in bronchial epithelium showed correlation with decreased CFTR expression [130]. 

Furthermore, miR-101 and miR-494 seem to act synergistically on CFTR-reporter inhibition with a more than additive effect on the post-translational control, which could have a physiological relevance in the complex disease phenotypes observed in CF [131].

The hallmark of CF lung disease is chronic infection by *Pseudomonas aeruginosa* that gradually increases from childhood through early adolescence. Rao et al. detected miRNAs in *P. aeruginosa* infected sputum of CF patients. A significant change in miR-146 expression in these patients was associated with the Toll-like receptor family, a family which includes the primary evolutionarily conserved sensors of pathogen-associated molecular patterns and is known to trigger host inflammatory and immune responses [132].

CF affects epithelial organs including the intestine, where both meconium ileus and distal intestinal obstruction syndrome can occur as complications. Bazett et al. [125] investigated whether miRNAs contribute to the different phenotypic changes observed in the CF intestine by initially measuring the miRNA signature of this tissue with an array. They concluded that altered miRNA expression is a feature that putatively influences both metabolic abnormalities and the altered tissue homeostasis component of CF intestinal disease [122].

The fact that a miRNA-regulated network directs gene expression from chromosome to cell membrane indicates that one individual miRNA can control a cellular process more broadly than recognized previously. This discovery will provide therapeutic avenues for restoring CFTR function to cells affected by the most common cystic fibrosis mutation and mandates miRNA-based research in this field [129].

## 6. Conclusion

Despite the inherent limitations, much progress has been made towards developing effective treatments for pediatric chronic diseases, offering hope for millions of children with these disorders. The role of miRNAs in the pathogenesis of these diseases makes them promising targets worth studying if our goal is to secure normal growth and development. Research efforts directed towards a greater understanding of the mechanisms and functional significance of the aberrant expression of miRNAs in these major chronic non-neoplastic diseases will assist in the development of less toxic therapies and provide better markers for disease classification. We believe that the discovery of miRNAs will open new research avenues for pediatric chronic diseases, which are expected to advance this area of research from its infancy to the mature stages.

## Figures and Tables

**Table 1:** An overview of miRNAs in the major chronic non-neoplastic childhood diseases.

Disease	miRNAs	Mechanism	Reference
*Bronchial asthma *			
(1) Risk	MiR-148a, miR-148b, and miR-152	Interacting with HLA-G	[9]
	pre-miRNAs	rs2910164G/C and rs2292832C/T SNP	[10]
	MiR-155	Decreased expression increase asthma severity	[11]
(2) Pathogenesis	MiR-146b, miR-223, miR-29b, miR-29c, miR-483, miR-574, miR-5p, miR-672, and miR-690	Abnormally expressed in asthma models	[12–19]
	MiR-221	Regulate mast cell functions	[20, 21]
	MiR-21	Polarize Th cells toward Th2	[12]
	MiR-126	Its blockage diminished Th2 responses	[13]
	MiR-146a	Contribute in remodeling	[25]
	let-7 mimic	Reduced IL-13 levels	[26]
	MiR-145	Pro-inflammatory effect	[27]
(3) Therapeutic targets	MiR-133a	Modulate RhoA/Rhokinase pathway	[17]
	MiR-126	Suppress Th2-driven airway inflammation	[13]
	MiR-106a	Inhibit IL-10	[35]
	MiR-146a	Mediate anti-inflammatory effect of dexamethasone	[36]
	Anti-miR-145	Reduce severity of airway inflammation	[37]

*Diabetes mellitus *			
(1) Physiological aspects			
(a) Pancreas development	MiR-124a2	Pancreatic *β*-cell development	[41]
	MiR-375	Formation of pancreatic islets	[42]
	MiR-375	Maintenance pancreatic endocrine mass viability	[43]
(b) Insulin biosynthesis	MiR-15a	Targeting UCP-2	[44]
	MiR-30d	Activates MafA expression	[45]
	MiR-375, miR-122, miR-127-3p, and miR-184	Insulin biosynthesis	[46]
	MiR-133a	Suppress insulin biosynthesis	[47]
(c) Insulin secretion	MiR-9	Secretory function of insulin producing cells	[48, 49]
	MiR-375	Regulate insulin secretion	[50]
	MiR-124a and miR-29	Optimal insulin secretion	[41, 52]
	MiR-33a	Inversely correlates with ABCA1 expression	[89]
	MiR-21, miR-34a, and miR-146	Inhibit insulin secretion	[54]
(d) Insulin actions	MiR-103/107	Insulin sensitivity	[55]
	Lin28/let-7	Regulation of glucose metabolism	[56]
(2) Type 1 diabetes	MiR-29 family	Cytokine-mediated *β*-cell dysfunction	[59]
	MiRs (124, 128, 192, 194, 204, 375, 672, and 708)	Deregulated in T1D model	[61]
(3) Type 2 diabetes	MiR-143	Inhibit insulin-stimulated AKT activation	[68]
	miR-146a impairment	Mediate insulin resistance	[69]
	MiR-125a	Increased expression in T2D	[70]
	MiR-126	Deregulated in plasma of T2D patients	[77]
(4) Complications	MiRs (144, 146a, 150, 182, 192, 30d, and 320)	Biomarkers for diabetes progression	[76]
	MiR-192	Increased in glomeruli of diabetic mice	[78]
	MiR-200b/c, miR-216a, and miR-217	Detected in glomeruli of diabetic mice	[79–81]
	MiR-377	Play a role in DN renal fibrosis	[82]
	MiR-192	Reduced renal fibrosis and improves proteinuria	[89]
	MiR-126, miR-27b, and miR-130a	Proangiogenic miRNAs	[89]
	MiR-98	Modulate TRB2	[90]
	MiR-503	Caused diabetic impaired angiogenesis	[91]
(5) Therapeutic targets	MiR-126	Related to impaired (EPC)	[92]
	MiR-186, miR-199a, and miR-339	Stem cell therapy of TID	[93]
	MiR-21-PDCD4 pathway	Treating autoimmune T1D	[94]
	MiR-375	Facilitate insulin response	[42]
	MiR-181a	Improves hepatic insulin sensitivity	[96]

*Epilepsy *			
(1) Pathogenesis	MiR-213, miR-132, miR-30c, miR-26a, and miR-375	Prominently upregulated in MTLE acute stage	[102]
	MiR-29a and miR-181c	Prominently downregulated in MTLE acute stage	[102]
	MiR-21	Regulate neurotrophin-3 signaling	[103]
	MiR-let-7e and miR-23 a/b	Deregulated in the MTLE chronic stage	[103]
	MiR-146a	Differently expressed in different stages of MTLE development and may interact with IL-1*β*	[107]
	MiR-155	Differently expressed in different stages of MTLE development and may interact with TNF-*α*	[108]
	MiR-132	Related to synaptic plasticity	[115]
(2) Potential blood biomarker	MiR-34a, miR-22, miR-125a, and miR-21	Showed different expression in the blood	[102]
(3) Therapeutic target	Anti-miR-132	Reduced seizure-induced neuronal death	[117]
	MiR-134 silencing	Neuroprotective effect	[118]

*Cystic fibrosis *			
	MiR-155	Activation of IL-8-dependent inflammation	[126]
	MiR-138	Regulates CFTR expression	[129]
	MiR-145, -223, and -494	Correlates with decreased CFTR expression	[130]
	MiR-101 and miR-494	Act synergistically on CFTR-reporter inhibition	[131]
	MiR-146	Significantly changed in the sputum of CF patients	[132]
